# Clinical application of RUBCN/SESN2 mediated inhibition of autophagy as biomarkers of diabetic kidney disease

**DOI:** 10.1186/s10020-022-00580-8

**Published:** 2022-12-07

**Authors:** Mona M. Watany, Hemat E. El-Horany, Marwa M. Elhosary, Ahmed A. Elhadidy

**Affiliations:** 1 Clinical Pathology Department, Faculty of Medicine, Tanta University, El Geish Street, Tanta, 31527 El-Gharbia Governorate Egypt; 2 Medical Biochemistry Department, Faculty of Medicine, Tanta University, Tanta, 31527 Egypt; 3 Msc Immunology from Tanta Faculty of Science, Tanta, 31527 Egypt; 4 Internal Medicine Department, Faculty of Medicine, Tanta University, Tanta, 31527 Egypt; 5 Biochemistry Department, College of Medicine, Ha’il University, Ha’il, 55211 Saudi Arabia

**Keywords:** Diabetic nephropathy, Autophagy, Mammalian/mechanistic target of rapamycin (mTOR), Rubicon (RUBCN), Sestrin-2 (SESN2), Insulin resistance, glycemic control, Diabetic kidney disease

## Abstract

**Background:**

Deregulated autophagy in diabetes has been a field of many experimental studies recently. Impaired autophagy in diabetic kidneys orchestrates every step of diabetic nephropathy (DN) pathogenesis. This study aimed to evaluate three autophagy regulators; RUBCN, mTOR, and SESN2 as clinically applicable indicators of DN progression and as early predictors of DN.

**Methods:**

This retrospective study included 120 participants in 4 groups; G1: diabetic patients without albuminuria, G2: diabetic patients with microalbuminuria, G3: diabetic patients with macroalbuminuria and G4: healthy controls. *RUBCN* and *SESN2* genes expression were tested by RT-qPCR. RUBCN, mTOR, and SESN2 serum proteins were quantitated by ELISA.

**Results:**

*RUBCN* mRNA was over-expressed in diabetic patients relative to controls with the highest level found in G3 followed by G2 then G1; (9.04 ± 0.64, 5.18 ± 0.73, 1.94 ± 0.41 respectively. P < 0.001). *SESN2* mRNA expression was at its lowest level in G3 followed by G2 then G1 (0.1 ± 0.06, 0.48 ± 0.11, 0.78 ± 0.13 respectively. P < 0.001). Similar parallel reduction in serum SENS2 was observed. Serum RUBCN and mTOR were significantly elevated in diabetic patients compared to controls, with the increase parallel to albuminuria degree. *RUBCN* expression, serum RUBCN and mTOR strongly correlated with albuminuria (r = 0.912, 0.925 and 0.867 respectively). *SESN2* expression and serum level negatively correlated with albuminuria (r = − 0.897 and -0.828 respectively); (All p < 0.001). Regression analysis showed that serum RUBCN, mTOR, *RUBCN* and *SESN2* mRNAs could successfully predict DN.

**Conclusions:**

The study proves the overexpression of RUBCN and mTOR in DN and the down-expression of SESN2. The three markers can be clinically used to predict DN and to monitor disease progression.

**Supplementary Information:**

The online version contains supplementary material available at 10.1186/s10020-022-00580-8.

## Background

Diabetic nephropathy (DN) is the most common cause of end-stage renal disease (ESRD). Diabetic kidney disease (DKD) starts with microalbuminuria that progress gradually to macro-albuminuria with concomitant deterioration of renal functions. Autophagy is a highly conserved cyto-protective process of physiological cellular organelles recycling and lysosomal degradation of macromolecules (Choi et al. 2013). Autophagy is induced by many intracellular stressors, such as hypoxia, starvation, reactive oxygen species (ROS), and endoplasmic reticulum (ER) stress (Tanaka et al. 2012).

In kidneys, autophagy is essential to maintain homeostasis of both renal glomeruli and tubules. In DN, autophagy is offended by the hyperglycemia related mitochondrial dysfunction, activation of protein kinase C (PKC), hypoxia, redox imbalance, endoplasmic reticulum stress, accumulation of advanced glycation end-products (AGE) and renin–angiotensin system activation. Additionally, hyperglycemia directly activates the mTOR pathway which negatively regulates autophagy (Ding and Choi 2015). The response to this impaired autophagic activity is induction of apoptosis, inflammation, and extracellular matrix (ECM) accumulation; that would be reflected as progression of albuminuria, decreased glomerular filtration rate (GFR), and eventually ends with the common response to injury, renal fibrosis and failure (Ding and Choi 2015).

Rubicon autophagy regulator (RUBCN) is a Beclin-1 interacting protein. RUBCN suppresses autophagy as it inhibits autophagosome maturation through binding to Beclin-1 pre-complexed with VPS34, VPS15, and UVRAG. The latter complex is essential for vesicle nucleation and phagophore formation (Ravikumar et al. 2010).

mTOR (mammalian/mechanistic target of rapamycin) is the serine/threonine kinase part of mammalian target of rapamycin complex 1 and 2 (mTORC1 and mTORC2). mTOR acts as a molecular link between cellular growth signals via phosphorylation of ribosomal S6Kinase (S6K) and eukaryotic translation initiation factor 4E-binding protein (4E-BP) (Howell et al. 2013). mTORC1 stimulates protein synthesis and increases cell size. mTORC2 manipulates aging, cell survival and cytoskeletal organization via phosphorylation of AKT, PKC, MST1, FOXO1, and cell cycle regulator (Gödel et al. 2011; Gui and Dai 2020). The active part of both molecules is the mTOR which is involved in almost every step of DKD pathogenesis (Yasuda-Yamahara et al. 2021).

mTORC1 is known to block the initial steps of autophagosome formation (Mizushima 2010). Moreover, mTORC1 contributes to insulin resistance as it suppresses adaptor proteins that acts downstream of insulin receptors and insulin-like growth factor receptors. mTORC1 is hyper-activated under glucose excess in many tissues including kidneys and is thought to be involved in the pathogenesis of DKD including podocytes damage, which causes proteinuria, and tubular cell injury resulting in decreased renal function (Yasuda-Yamahara et al. 2021). Recently, the role of mTORC2 in regulating pancreatic β-cell mass, survival and function under diabetogenic conditions has been elucidated. Selective inactivation of mTORC2 in β cells results in reduction of β-cell mass, proliferation and glucose-induced insulin secretion (Yuan et al. 2018). Data about mTORC2 role in DKD is controversial and mTORC2 activity control is less well understood than mTORC1, although growth factors appear to stimulate both complexes (Gödel et al. 2011; Grahammer et al. 2014; Lieberthal and Levine 2012a).

Sestrin-2 (SESN2) is a member of a highly conserved family of stress responsive proteins that have antioxidant properties. Sestrins expression is induced by environmental and metabolic noxious stimuli, like oxidative stress, inflammation and DNA damage, in order to maintain cellular homeostasis and survival. Beside SESN2 function as suppressor of ROS formation, it inhibits the activity of mTORC1. Hence, SESN2 is a positive autophagy regulator (Budanov and Karin 2008; Lin et al. 2020).

Although accumulating reports suggest microalbuminuria as a risk factor for developing nephropathy and macroalbuminuria, not all patients progress to this stage and some patients may even regress to normoalbuminuria state. Hence there is a need for earlier and sensitive markers to predict and monitor nephropathy in diabetic patients. The aim of this work was to evaluate three autophagy regulators; Rubicon (RUBCN), mTOR and Sestrin-2 (SESN2) as clinically applicable biomarkers of diabetic nephropathy; and to assess if they can be used to predict the development of nephropathy in diabetic patients.

## Methods

### Patients selection and ethical approval

The current study included 120 participants categorized in four groups, each group included 30 participants. Group 1 (G1): diabetic patients with normal urinary albumin excretion (< 30 mg albumin/g creatinine), group 2 (G2): diabetic patients with micro-albuminuria (30–300 mg albumin/g creatinine), group 3 (G3): diabetic patients with macro-albuminuria (> 300 mg albumin/g creatinine) and group 4 (G4): healthy controls. Diabetic patients were enrolled from the Endocrinology and Diabetes Outpatient Clinics of Tanta University Hospital. Patients with chronic infections, concomitant liver diseases, cardiovascular diseases, malignancies, autoimmune diseases and other renal diseases than DN, were excluded. The study complies with Helsinki’s declaration and was approved by local ethical review board Faculty of Medicine, Tanta University. An informed written consent was obtained from all of the participants. The healthy controls were volunteers of matched sex and with the same age range as the participants. Diabetic patients with and without nephropathy were receiving their hypoglycemic medications; but none of them was receiving any treatment for albuminuria.

### Samples collection and storage

Blood samples were drawn under the standard infection control guidelines. Fasting serum samples were immediately used for biochemical analysis of glucose, cholesterol, triglycerides, HDL-cholesterol and insulin level. An aliquot was stored at − 80 °C for measurements of RUBCN, mTOR, and SENS2 by ELISA. To separate sera, samples were allowed to clot at room temperature then, centrifuged at 1000×*g* for 20 min. Samples showing hemolysis were rejected. Two separate aliquots of K3EDTA blood were also drawn; one for HbA1C estimation and the other for RNA extraction. For albumin/creatinine ratio (ACR) estimation, second morning urine fresh samples were collected for measurements of urinary albumin and urinary creatinine.

### Routine laboratory investigations

Glucose, cholesterol, triglycerides, HDL-cholesterol, creatinine and HbA1C were measured using automated chemistry analyzer (Beckman coulter AU48, Siemens, USA). Fasting insulin was measured by chemiluminescence immunoassay analyzer Cobas® e411 (Hoffmann La Roch Ltd, Switzerland). LDL-cholesterol was calculated according to Friedewald’s formula. Homeostatic Model Assessment of Insulin Resistance (HOMA-IR) was calculated from the formula: fasting insulin (microU/L) × fasting glucose (nmol/L)/22.5. For ACR, Urinary albumin was quantitated using immune-turbidmetric method by a commercially available kit (Biosystems, Spain). All of the kits and instruments used are IVD approved.

### RUBCN, mTOR and SENS2 measurement by ELISA

Serum Rubicon was measured using Human Rubicon (RUBCN) ELISA Kit (Catalog No: abx259826, Abbexa LTD, Cambridge, UK). The kit assay range is: 0.312–20 ng/mL and it is sensitive down to 0.124 ng/mL. Serum mTOR was measured using a Human mTOR (Mammalian Target of Rapamycin) ELISA Kit (Catalog No: MBS2505637, Mybiosource, San Diego, California, USA). The kit has a detection range of 0.16–10 ng/mL and an analytical sensitivity of 0.1 ng/mL. Serum SESN2 was measured by Human SESN2 (Sestrin-2) ELISA Kit, (Catalogue No.: EH1556, FineTest®, Wuhan, China). The kit detection range is: 0.156–10 ng/mL with the low limit of detection of 0.094 ng/mL. All ELISA assays were performed according to the manufacturers’ instructions. The optical density of the final yellow color was measured using ELISA Spectrophotometry Reader (Stat Fax 2100, Fisher Bioblock Scientific, France). Curve expert 1.4 basic software was used to generate the standard curves and to calculate the concentrations of the samples from their corresponding optical density.

### RUBCN and SESN2 relative gene expression by RT-qPCR

RNA was extracted from fresh k3-EDTA blood using QIAamp RNA blood mini kit (cat no. 52304, Qiagen, Gmbh, Germany) with on-Column DNase digestion by RNase-Free DNase Set. RNA concentration and quality were checked using NanoDrop 1000 drop (Thermo Fisher Scientific, USA). RNA was converted to cDNA with reverse transcriptase, (Power cDNA Synthesis Kit (First-strand cDNA Synthesis), Cat. No. 25011, iNtRON Biotechnology, Korea), according to manufacturers’ instructions. Two micrograms of total RNA were used for each reaction and the cDNA was stored at − 80 °C in cryotubes for no longer than 3 months. RT-qPCR was used to evaluate the expression of RUBCN and SESN2 relative to ß-Actin (*ACTB*) as a reference gene after validation of the latter. The sequences of the utilized primers are shown in Additional file 1: Table S1. Each PCR reaction contained about 30 ng of cDNA, 1uM of each primer and 12.5 µl of 2X Quantifast SYBR Green PCR Master Mix (Qiagen, Germany) in a total reaction volume of 25 µl. Non template controls were included in each run to monitor any contamination. To monitor technical viabilities, 3 samples replicates were amplified blindly within each run. Step One® Real–Time PCR System (Applied Biosystems, USA) was used for amplification and relative quantitation of RUBCN and SESN2 mRNA expression relative to β-actin using 2^−∆∆CT^ method. The thermal profile was as the manufacturer instructed; 1 cycle at 95 °C for 5 min to activate DNA polymerase, then 40 cycles of (denaturation at 94 °C for 10 s and Combined annealing/extension at 60 °C for 30 s). The process was followed by melting curve analysis to ensure the specificity and identity of PCR product.

### Statistical analysis

All statistical tests were carried out using SPSS for windows (version 19, IBM, USA). The reported findings passed Shapiro Wilk test for normal distribution. Numerical data are reported as means and standard deviations (SD). Non-numerical data are presented as frequencies (n) and percentages (%). The means are compared with analysis of variance (ANOVA) test followed by post hoc Tukey’s test. Frequencies are compared with (Chi-square) test. Pearson correlations were run to evaluate the relationship between the tested autophagy markers, glycemic control and albuminuria. Regression analysis was used to evaluate the ability of the studied markers to predict nephropathy in diabetic patients. P values less than 0.05 was taken as the level of significance (alpha error set to 5% at 95% confidence interval). ROC curve analysis was performed for the studied markers to evaluate their clinical diagnostic performance as early biomarkers of DN. The power of the study was also computed using alpha = 0.05; partial Eta squared values were 0.903, 0.820, 0.920, 0.893 and 0.936 for serum RUBCN, serum SESN2, serum mToR, RUBCN expression and SESN2 expression respectively. The observed powers were 100%.

## Results

The four studied groups showed statistically significant differences between the measured levels of HbA1C, Cholesterol, triglycerides, serum creatinine as well as the calculated body mass index (BMI), Homeostatic Model Assessment of Insulin Resistance (HOMA-IR), LDL- cholesterol and albumin/creatinine ratio (ACR). HDL-cholesterol did not significantly differ between the 4 groups. Those differences are shown in details in Table 1.

**Table 1:** The demographic and laboratory findings of the studied 120 participants

	Group 1 Diabetes without nephropathy (N. = 30)	Group 2 Diabetes with microalbuminuria (N. = 30)	Group 3 Diabetes with macro-albuminuria (N. = 30)	Group 4 Healthy controls (N. = 30)	F/chi^2^ when appropriate	Sig
Age (years; mean ± SD) [range, years]	40.13 ± 7.02 [33–55]	49.43 ± 10.31 [38–60]	50.43 ± 8.18 [40–62]	44.5 ± 10.34 [33–62]	13.93	0.453
Time from initial diagnosis	2.1 ± 0.8^c,d^	4.3 ± 1.4^b,d^	5.2 ± 1.8^b,c^	–	22.36	0.002*
Sex						
Male (n;%)	16 (53%)	14 (47%)	14 (47%)	15 (50%)	0.533	0.912
Female (n;%)	14 (47%)	16 (53%)	16 (53%)	15 (50%)		
BMI	23.9 ± 2.1^a,c,d^	26.4 ± 1.8^b^	26.7 ± 2.3^b^	25 ± 1.8^b^	11.39	< 0.001*
HOMA-IR	2.65 ± 0.32^a,c,d^	2.89 ± 0.27^a,b,d^	3.16 ± 0.37^a,b.c^	1.28 ± 0.25^b,c,d^	224.16	< 0.001*
HbA1C	6.32 ± 0.71^a,c,d^	7.27 ± 0.63^a,b,d^	8.51 ± 0.85^a,b,c^	2.44 ± 0.71^b,c,d^	386.69	< 0.001*
Cholesterol (mmol/L)	6.23 ± 0.84^a^	5.98 ± 0.46^a,d^	6.67 ± 1.02^b^	5.02 ± 0.74^b,c,d^	23.401	< 0.001*
Triglycerides (mmol/L)	2.16 ± 0.35^a^	1.96 ± 0.46^a,d^	2.37 ± 0.57^a,c^	1.53 ± 0.34^b,c,d^	20.121	< 0.001*
HDL-Cholesterol (mmol/L)	1.23 ± 0.32	1.25 ± 0.33	1.14 ± 0.32	1.23 ± 0.24	0.863	0.462
LDL-Cholesterol (mmol/L)	4.01 ± 0.86^a^	3.28 ± 0.49^a,d^	4.45 ± 1.01^a,c^	3.09 ± 0.81^b,c,d^	14.554	< 0.001*
Creatinine (µmol/L)	83.98 ± 13.26^d^	85.75 ± 13.26^d^	114.92 ± 15.91^a,b,c^	88.40 ± 14.14^d^	33.2	< 0.001*
ACR	19.67 ± 4.23^c,d^	129.67 ± 34.92^a,b,d^	383.77 ± 54.72^a,b,c^	15.37 ± 2.54^c,d^	844.924	< 0.001*

ANOVA analysis with post hoc Tukey’s test; (*) indicates statistical significance), data is presented as mean ± Standard deviation

^a^Significant p value (< 0.05) compared to controls (group 4)

^b^Significant p value (< 0.05) compared to group 1

^c^Significant p value (< 0.05) compared to group 2

^d^Significant p value (< 0.05) compared to group 3

BMI: body mass index; HOMA-IR: Homeostatic Model Assessment of Insulin Resistance; HbA1C; glycated hemoglobin; HDL-Cholesterol: high density lipoproteins-cholesterol; LDL-Cholesterol: low density lipoproteins-cholesterol; ACR: albumin/creatinine ratio in urine

Conversion of traditional units into SI unit: cholesterol: multiply concentration in mg/dL × 0.0259; creatinine: multiply concentration in mg/dL × 88.4; triglycerides: multiply concentration in mg/dL × 0.0113

Regarding serum RUBCN, the highest levels were found in G3 (mean = 6.75 ± 1.12 ng/mL), followed by G2 (4.05 ± 0.88 ng/mL), then by G1 (1.54 ± 0.18 ng/mL). Serum levels of RUBCN did not significantly differ between controls (G4) and diabetic patients without nephropathy (G1) (Table 2A).

**Table 2:** Comparison between RUBCN, SESN2, mTOR serum levels, *RUBCN* and *SESN2* expression among the studied groups

	Group 1 Diabetes without nephropathy (N. = 30)	Group 2 Diabetes with microalbuminuria (N. = 30)	Group 3 Diabetes with macroalbuminuria (N. = 30)	Group 4 Healthy controls (N. = 30)	F	Sig
(A) Serum levels of RUBCN, SESN2and mTOR and relative RUBCN and SESN2 mRNA expression						
RUBCN serum level (ng/mL)	1.54 ± 0.18 ^c,d^	4.05 ± 0.88 ^a,b,d^	6.75 ± 1.12 ^a,b,c^	1.48 ± 0.17	359.523	< 0.001*
SESN2 serum level (ng/mL)	6.47 ± 0.86 ^a,c,d^	5.27 ± 0.61 ^a,b,d^	4.02 ± 0.47 ^a,b,c^	8.04 ± 0.76	221.661	< 0.001*
mTOR serum level (ng/mL)	2.5 ± 0.5 ^a,c,d^	4.05 ± 0.88 ^a,b,d^	6.11 ± 0.6 ^a,b,c^	0.65 ± 0.17	441.866	< 0.001*
*RUBCN* expression	1.94 ± 0.41 ^a,c,d^	5.18 ± 0.73 ^a,b,d^	9.04 ± 0.64 ^a,b,c^	Ref	330.755	< 0.001*
*SESN2* expression	0.78 ± 0.13 ^a,c,d^	0.48 ± 0.11 ^a,b,d^	0.1 ± 0.06 ^a,b,c^	Ref	563.214	< 0.001*
(B) Normalized serum levels of RUBCN, SESN2 and mTOR to total serum proteins						
RUBCN (ng/gm protein)	22.81 ± 2.91 ^c,d^	63.18 ± 15.68 ^a,b,d^	122 ± 26.58 ^a,b,c^	21.82 ± 2.3	277.7	< 0.001*
SESN2 (ng/gm protein)	110.62 ± 13.54 ^a,c,d^	81.73 ± 10.53 ^a,b,d^	37.99 ± 10.8 ^a,b,c^	118.8 ± 12.42	101.8	< 0.001*
mTOR (ng/gm protein)	36.98 ± 7.67 ^a,c,d^	62.78 ± 13.69 ^a,b,d^	112.5 ± 13 ^a,b,c^	9.66 ± 4.04	532.8	< 0.001*

ANOVA analysis with post hoc Tukey’s test; (*) indicates statistical significance), data is presented as mean ± Standard deviation

^a^Significant p value (< 0.05) compared to controls (group 4)

^b^Significant p value (< 0.05) compared to group 1

^c^Significant p value (< 0.05) compared to group 2

^d^Significant p value (< 0.05) compared to group 3

RUBCN: Rubicon; SESN2: Sestrin-2; mTOR: mammalian target of Rapamycin

In contrast to RUBCN, even in the absence of nephropathy, diabetic patients from G1 showed lower levels of SESN2 compared to healthy controls (6.47 ± 0.86 ng/mL and 8.04 ± 0.76 ng/mL respectively). The lowest serum SESN2 level was observed in G3 (4.02 ± 0.47 ng/mL) followed by G2 (5.27 ± 0.61 ng/mL), then by G1 (Table 2A).

Serum mTOR levels were higher in diabetic patients compared to controls with the highest levels found in G3 followed by G2 then by G1 (6.11 ± 0.6, 4.05 ± 0.88 and 2.5 ± 0.5 respectively). Even in the absence of nephropathy, diabetic patients from G1 showed higher levels of mTOR compared to healthy controls (Table 2A). Upon normalization to total serum proteins the same significant differences were found in between the 4 studied groups (Table 2B).

*RUBCN* mRNA expression was increased several folds in diabetic patients compared to controls; with an obvious relation to albuminuria degree. The highest levels observed in G3 followed by G2 then by G1 (9.04 ± 0.64, 5.18 ± 0.73 and 1.94 ± 0.41 respectively). A marked reduction of *SESN2* expression was observed in the presence of nephropathy with the lowest levels observed in the presence of macro-albuminuria. For both genes transcripts, post hoc test showed that each group was statistically different from the other groups (all *P* were < 0.01) (Table 2A).

RUBCN serum level and mRNA expression showed strong positive correlations with serum mTOR and negatively correlated with SESN2. Higher levels of RUBCN were associated with poor glycemic control (higher HbA1C) and higher ACR (Table 3).

**Table 3:** Correlation matrix between the studied autophagy markers, HOMA-IR, HbA1C and the degree of albuminuria

	Serum RUBCN	serum SESN2	serum mTOR	*RUBCN* expression	*SESN2* expression
	*r*	*r*	*r*	*r*	*r*
HOMA IR	0.670*	− 0.733*	0.825*	0.751*	− 0.798*
HbA1C	0.707*	− 0.746*	0.859*	0.791*	− 0.830*
ACR	0.925*	− 0.830*	0.867*	0.946*	− 0.897*
Serum RUBCN	1	− 0.863*	0.854*	0.940*	− 0.902*
serum SESN2	− 0.863*	1	− 0.859*	− 0.895*	0.872*
serum mTOR	0.854*	− 0.859*	1	0.923*	− 0.922*
*RUBCN* expression	0.940*	− 0.895*	0.923*	1	− 0.948*
*SESN2* expression	− 0.902*	0.872*	− 0.922*	− 0.948*	1

^*^indicates statistical significance (*P* < 0.05); *r* = Pearson correlation coefficient. All calculated *P* were significant < 0.001

HOMA-IR: Homeostatic Model Assessment of Insulin Resistance; HbA1C; Glycated hemoglobin; ACR: Albumin/Creatinine ratio in urine; RUBCN: Rubicon; SESN2: Sestrin2; mTOR: mammalian target of Rapamycin

Pearson’s correlation test was used

SESN2 serum level and mRNA expression negatively correlated with serum mTOR, serum RUBCN and *RUBCN* expression. SESN2 negatively correlated with HbA1C and ACR (Table 3).

Multiple regression analysis was run to predict microalbumiuria from RUBCN transcript and protein, mTOR and SESN2 transcript and protein. The variables successfully passed the check for suitability of the model (F = 165.1, *p* < 0.001, adjusted R^2^ = 0.902). Of course some multicolinearity was observed within the model especially between each protein and its mRNA transcript. Serum RUBCN, serum mTOR, *RUBCN* expression and *SESN2* expression were able to predict albuminuria (*P* < 0.001, 0.003, < 0.001 and 0.019 respectively) while serum SESN2 was not able to predict it (*P* 0.09) (Table 4).

**Table 4:** Linear regression analysis test results for evaluation of the ability of serum RUBCN, serum SESN2, serum mTOR, *RUBCN* mRNA expression and *SESN2* mRNA expression to predict nephropathy in diabetic patients

Model	Unstandardized coefficients		Standardized coefficients	t	Sig
	B	Std. Error	Beta		
(A) simple linear regression analysis					
(Constant)	− 77.284	9.729		− 7.944	< 0.001*
Serum RUBCN	62.049	2.350	0.925	26.403	< 0.001*
(Constant)	582.634	28.636		20.346	< 0.001*
Serum SESN2	− 71.858	4.442	− 0.830	− 16.177	< 0.001*
(Constant)	− 73.796	13.168		− 5.604	< 0.001*
Serum mTOR	63.356	3.348	0.867	18.922	< 0.001*
(Constant)	− 57.630	7.626		−7.557	< 0.001*
RUBCN expression	45.475	1.429	0.946	31.820	< 0.001*
(Constant)	368.818	12.238		30.136	< 0.001*
SESN2 expression	− 393.326	17.878	− 0.897	− 22.001	< 0.001*
(B) multiple linear regression analysis					
(Constant)	91.198	68.350		1.334	0.185
Serum RUBCN	27.094	5.557	0.407	4.876	< 0.001*
Serum SESN2	3.044	5.619	0.153	1.580	0.09
Serum mTOR	19.946	6.550	0.273	3.045	0.003*
*RUBCN* expression	12.926	3.791	0.274	3.409	< 0.001*
*SESN2* expression	− 103.833	43.678	− 0.238	− 2.377	0.019*

Dependent variable: Albumin/creatnine ratio

^*^Indicates statistical significance

RUBCN: Rubicon; SESN2: Sestrin-2; mTOR: mammalian target of Rapamycin

Multiple regression test was used to assess the ability to predict Albumin/creatnine ratio based on the level of serum RUBCN, serum SESN2, serum mTOR, *RUBCN* mRNA expression and *SESN2* mRNA expression

Higher *RUBCN* expression above 3.4 folds of normal was able to identify DN with 100% sensitivity and specificity. Decreased expression of *SESN2* mRNA below 0.65 folds of normal was able to identify DN with 98% sensitivity and 83.3% specificity.

**Fig. 1:**
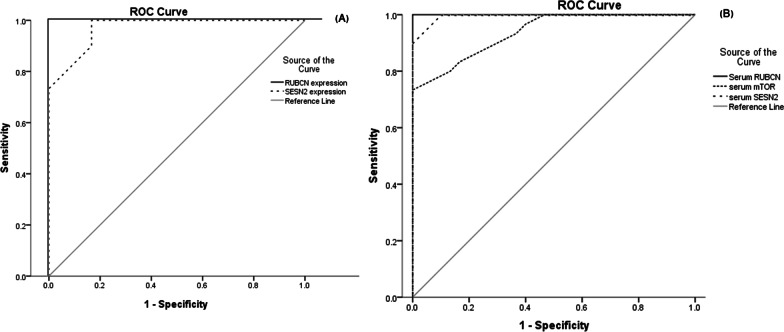
Receiver operating characteristics curve of the studied proteins and transcripts as biomarkers of Diabetic nephropathy. **A** ROC curve of RUBCN and SESN2 transcripts as diagnostic biomarkers of diabetic nephropathy. **B** ROC curve of serum RUBCN, SESN2 and mTOR as diagnostic biomarkers of diabetic nephropathy

RUBCN serum levels above 2 ng/mL were able to diagnose DN with 100% sensitivity and 98% specificity. Serum mTOR above 3.05 ng/mL was able to differentiate DN with 83.3% sensitivity and 84.3% specificity. Serum SESN2 lower than 6.15 ng/mL could differentiate DN with 96.7% sensitivity and 94.3% specificity. RUBCN performance was strikingly impressive (Table 5 and Fig. 1).

**Table 5:** ROC curve analysis of serum RUBCN, SESN2, mTOR, and *RUBCN* and *SESN2* expression as biomarkers of early nephropathy

Test Result Variable(s)	ROC_AUC_ (95% CI)	Cutoff	Sensitivity (%)	Specificity (%)	Std. Error	Asymptotic Sig
Serum RUBCN (ng/mL)	1	> 2	100	98	0.000	< 0.001*
Serum SESN2 (ng/mL)	0.995 (0.985–1)	< 6.15	96.7	68.5	0.047	< 0.001*
Serum mTOR (ng/mL)	0.937 (0.881–0.993)	> 3.05	83.3	84.3	0.029	< 0.001*
Relative *RUBCN* expression	1	> 3.4	100	100	0.000	< 0.001*
Relative *SESN2* expression	0.969 (0.935–1)	< 0.65	98	83.3	0.017	< 0.001*

^*^Indicates statistical significance (P < 0.05); All calculated *P* were < 0.001

ROC: receiver operating characteristics; AUC: area under the curve; CI: confidence interval; RUBCN: Rubicon; SESN2: Sestrin-2; mTOR: mammalian target of rapamycin

Receiver operating curve was constructed using patients test results. The cutoff point was selected to be the closest to the upper left corner of the curve to achieve the highest sensitivity and specificity

## Discussion

In diabetes mellitus, autophagy instead of being induced by cellular stress and organelles dysfunction, is impaired due to alteration of nutrient sensing pathways like mammalian target of rapamycin (mTOR), Adenosin monophosphate-activated protein kinase (AMPK) and NAD^−^ dependent histone deacetylase (Sirt1) (Tanaka et al. 2012).

The inhibited autophagic activity in diabetic kidneys affects the whole nephron. On one hand, glomeruli suffer from podocytes loss, basement membrane thickening, endothelial cells dysfunction, mesangial cellular and matrix expansion, and glomerulosclerosis. On the other hand, proximal tubular epithelial cells suffer from cellular hypertrophy and eventual degeneration. The two arms reinforce each other during the development of diabetic nephropathy (Ding and Choi 2015).

The current study showed elevated *RUBCN* mRNA expression and serum protein in DN patients compared to controls, indicating poor autophagic activity in DKD since RUBCN is a negative regulator of autophagy. To the best of authors’ knowledge, this is the first study to measure *RUBCN* transcript and protein in DN patients’ peripheral blood.

Rubicon is a ubiquitously expressed protein found in most tissue and organs of vertebrate species. It acts as a negative regulator of canonical autophagy and endosomal trafficking and a key modulator of immune-tolerance, inflammatory response and viral replication. It has been shown to be implicated in aging, recessive ataxia, non-alcoholic fatty liver disease, cholestasis, systemic lupus erythematosus, inflammatory bowel diseases and in modulating HBV and HCV replication (Wong et al. 2018; Martinez et al. 2015).

Recently Li et al. studied *RUBCN* expression in podocytes cultures and in animal models. They proved that under high glucose levels, renal epidermal growth factor receptor (EGFR) pathway is activated and autophagic activity is diminished. This was evidenced by decreased beclin-1, inhibition of autophagosome formation and increased RUBCN. Moreover, Li et al. (2021) clearly elucidated that knocking down *RUBCN* prevents glucose-induced inhibition of autophagy in cultured podocytes. EGFR pathway activation leads to phosphorylation of tyrosine residues found in many signaling molecules, including the MAP kinase, JAK/STAT pathway and phosphatidyl-inositol-3-kinase (PI3K), which are involved in cellular proliferation, differentiation, insulin sensitivity, autophagy and apoptosis (Li et al. 2021; Sun et al. 2021). In similar context, Nakamura et al. (2019) observed that Rubicon knockout in mice is protective against interstitial kidney fibrosis.

Moreover, Matsuda et al. proved that RUBCN deficiency in cultured kidney proximal tubular epithelial cells enhances autophagy. But their experiment on *RUBCN*-deficient mice showed an unexpected association between RUBCN deficiency and impaired glucose tolerance as well as insulin resistance (Matsuda et al. 2020). In contrast, our study showed that RUBCN excess was associated with poor glycemic control (higher HbA1C) and with more insulin resistance (higher HOMA-IR).

In the current study elevated RUBCN correlated with albuminuria degree. This agrees with Tagawa et al. who clarified the crucial role of autophagy in maintaining podocytes’ lysosomal hemostasis under diabetic conditions. Impaired autophagy in diabetic podocytes aggravates their loss and leads to massive proteinuria in diabetic nephropathy models (Tagawa et al. 2016). Although Nakamura et al. (2019) suggested Rubicon expression increases age-dependently, such correlation could not be proved in the current study.

In the current study serum mTOR was significantly elevated in diabetic patients (with and without nephropathy) compared to controls. And this elevation correlated positively with HOMA-IR. These findings agree with Howell et al. (2013) and Hartman et al. (2009) who claimed that chronic activation of mTOR induces insulin resistance through inhibiting the phosphorylation of insulin receptor substrate-1 (IRS1) by mTORC1, and enhancing its proteosomal degradation by mTORC2. Additionally, Lee et al. (2013) demonstrated the inhibitory role of mTOR activation on insulin action via reduced activity of PI3K, the enzyme that promotes the insulin signaling.

Once diabetes develops, hyperglycemia creates a vicious circle of mTOR activation, inhibition of AMPK and deregulation of Akt pathway. This over-active mTOR is directly related to unbalanced glomerular epithelia hypertrophy, podocyte effacement then glomerulosclerosis which precede irreversible nephron’s loss of function (Grahammer et al. 2014; Lieberthal and Levine 2012b).

In this current study, serum mTOR was higher in DN patients compared to diabetic patients with normal urinary albumin excretion. Although no similar results are reported in humans to date, this finding generally agree with several studies carried out on animal models with induced diabetes. In 2006 Lloberas et al. and Sakaguchi et al. proved the effect of increased mTORC1 on diabetic kidneys. Blockade of the mTOR pathway in diabetic mice models suppressed DN development (Lloberas et al. 2006; Sakaguchi et al. 2006). It is documented that inappropriate mTORC1 over-activity contributes to metabolic syndrome progression, type 2 diabetes development, and DN pathogenesis (Lieberthal and Levine 2012b).

The current study showed Positive correlation between mTOR and proteinuria degree. Godel et al. (2011) and Inoki et al. (2011) proved the deleterious effect of mTORC1 hyperactivity on diabetic mice podocytes. They also found that suppression of mTORC1 activity can reduce urinary proteins excretion and prevent the progression of DKD. Since mTORC1 negatively regulates autophagy, its excess results in autophagy deficiency which causes sever podocytes damage and massive proteinuria (Tagawa et al. 2016).

Additionally, Tubular cells overwhelmed by proteinuria suffer from inflammation; and in the presence of abnormal mTORC1 excess, proteinuria- induced autophagy is inhibited (Yasuda-Yamahara et al. 2021). Moreover, mTOR pathway activation in diabetic kidneys induces interstitial fibrosis via increased expression of profibrotic cytokines, like tissue growth factor-1 (TGF-1) and connective tissue growth factor (CTGF). The end result will be tubular injury, interstitial fibrosis and decline of renal functions (Lloberas et al. 2006; Sakaguchi et al. 2006). Recently Tomita et al. showed that in mice models with DKD, tubular mTORC1 hyperactivity was associated with tubular damage and declined renal functions even in the absence of proteinuria (Tomita et al. 2020).

Additionally, the role of oxidative stress in the pathogenesis of DKD cannot be ignored. Hyperglycemia, increased free fatty acids, accumulation of AGE products, activation polyol and protein kinase C pathways and accumulation of damaged mitochondria, all induce ROS production (Yasuda-Yamahara et al. 2021; Wang and Klionsky 2011). Oxidative stress should induce autophagy to protect diabetic patients’ kidneys, but this does not occur in the presence of an inappropriate mTORC1 hyper-activation. There is a direct link between mTORC1 signaling and oxidative stress. Excessive mTORC1 activation induce insulin resistance which promotes ROS synthesis (Yasuda-Yamahara et al. 2021). This infinite loop is implicated in podocytes and tubular injury that occur in DN. In concordance with this context, in the current study, a positive correlation between mTOR and insulin resistance was found.

Eid et al. proved the effect of targeted inhibition of mTORC2 in mice models. It reduced podocytes loss and attenuated oxidant-mediated glomerular injury and albuminuria (Eid et al. 2016). Additionally, Das et al. (2018) elucidated the role of TGFβ in hyper-activation of both mTORC1 and mTORC2 in fibrotic kidney diseases like diabetic nephropathy.

ATP-competitive (catalytic) inhibitors of mTOR that can deactivate both mTORC1 and mTORC2 are currently used for some solid tumors as anti-cancer agents, and as immune-modulator after various organs transplantation to prevent virus associated nephropathy. The authors believe those inhibitors might offer new avenues for DN therapy.

SESN2 is a stress-inducible metabolic regulator. The protein structure is conserved throughout the metazoan species. Sestrins help cellular adaptation to stress stimuli. Its protective role has been described in many diseases like cardiomyopathy, atherosclerosis, diabetes, obesity, cancer and neurodegenerative disease like Parkinson’s disease and Alzheimer’s disease (Pasha et al. 2017; Kim et al. 2015).

The current study showed that both SESN2 gene expression and serum protein level decrease in parallel with the degree of albuminuria; with the lowest levels found in the presence of macroalbuminuria. This typically agrees with a study by Mohany and Rugaie (2020) who recently reported reduced SENS2 levels in diabetic patients’ sera and its negative correlation with the degree of albuminuria. Additionally, these findings are in concordance with Lin et al. who proved the down regulation of SESN2 in streptozotocin (STZ)-induced diabetes animal models. They elucidated SESN2 role in perturbed podocyte mitochondrial dysfunction under hyperglycemic conditions through the down regulation of AMPK (Lin et al. 2020). AMPK is a potent positive regulator of autophagy via inhibition of mTORC1 activity (Lee et al. 2010). It is worth mentioning that AMPK also has antioxidant properties and protects mitochondrial function (Madhavi et al. 2019).

The reduced sestrin-2 levels in diabetic patients compared to healthy controls agree with Li et al. (2017) and Catrina and Zheng (2021) who demonstrated the important role of sestrin-2 in maintaining insulin sensitivity via AMPK -dependent autophagy pathway activation. Those reduced SESN2 levels in diabetes were not found in Chung et al. study (Chung et al. 2018).

Under physiological conditions, SESN2 is up-regulated in response to cellular glucose scarcity, hypoxia, ER stress, oxidative stress and DNA damage (Seo et al. 2016). SESN2 is a key sensor for the mTORC1 pathway in mammalian cells to promote autophagy (Wolfson et al. 2016). It directly binds and functions upstream of AMPK to inhibit mTORC1 and induce autophagy in order to maintain cellular and physiological metabolic homeostasis (Budanov and Karin 2008; Liu et al. 2015). In diabetes mellitus, these cyto-protective mechanisms proved to be impaired. In the absence of SESN2, cells become highly susceptible to apoptosis, lipid accumulation, protein aggregate formation and mitochondrial dysfunction, a group of pathologies collectively known as ER-related pathologies. Those ER-related pathologies result in impaired autophagy (Chen et al. 2022).

In the current study, SESN2 negatively correlated with glycemic control. This agrees with the findings of Mohany and Rugaie (2020) and Sundararajan et al. (2021). This correlation can be explained by the protective effect of SESN2 against insulin resistance formerly described. Furthermore, as expected, SESN2 negatively correlated with mTOR. Similar relationship was demonstrated by Li et al. (2017).

## Conclusions

The current study adds to the accumulating body of evidences about autophagy inhibition in diabetes mellitus and its direct relation to the development of DN. Rubicon and mammalian target of rapamycin, the negative regulators of autophagy, are overexpressed in diabetic patients with nephropathy while, Sestrin-2 is down-expressed. The three tested markers can be clinically utilized in humans to predict DKD and monitor its progression. Moreover, the findings of this study might propose a rational for targeting autophagy as renal-protective therapy in diabetic patients. However, to authors’ knowledge, few studies on the three investigated markers RUBCN, mTOR and SESN2, have been published to date and the majority of these studies were on animal models. So, further studies on diabetic patients are encouraged. Also, it would serve as a proof of concept to test the studied genes and proteins by different methodologies and to compare the expression patterns utilizing different techniques.

## Supplementary Information

**Additional file 1: Table S1.** List of oligonucleotide primers.

## Data Availability

The data that support the findings of this study are available from the corresponding author upon reasonable request.

## Abbreviations

ACR: Albumin/creatinine ratio
AMPK: Adenosin monophosphate-activated protein kinase
DKD: Diabetic kidney disease
DN: Diabetic nephropathy
ECM: Extracellular matrix
ELISA: Enzyme-linked immuno-sorbent assay.
ER: Endoplasmic reticulum
ESRD: End stage renal disease
GFR: Glomerular filtration rate
mTOR: Mammalian/mechanistic target of rapamycin
PI3K: Phosphatidyl-inositol-3-kinase
PKC: Activation of protein kinase C
ROS: Reactive oxygen species
RUBCN: Rubicon
SESN2: Sestrin-2
