# Supplementary material for: Clinical application of RUBCN/SESN2 mediated inhibition of autophagy as biomarkers of diabetic kidney disease
Source: Mol Med. 2022 Dec 7;28:147. doi: 10.1186/s10020-022-00580-8 (PMC9730641; doi:10.1186/s10020-022-00580-8)
Supplement: Supplementary file 1 — Additional file 1: Table S1. List of oligonucleotide primers. [file 10020_2022_580_MOESM1_ESM.docx]

**Table S1**. List of oligonucleotide primers

| target | Primer sequence |
| --- | --- |
| RUBCN | F: 5’- TCCAGGAGGCCCCAGGAATA-3’  R: 5’- GCACAGGTACCGACCACCTT-3’ |
| SESN2 | F:5’- CTTCACTCGGAGAGGGGCTC-3’  R:5’- AGGGTAAAGCCGCTGGATCA-3’ |
| ACTB | F: 5’- GGCGGCAACACCATGTACCCT-3’  R:5’- AGGGGCCGGACTCGTCATACT-3’ |
